# World Trade Organization membership and changes in noncommunicable disease risk factors: a comparative interrupted time-series analysis, 1980–2013

**DOI:** 10.2471/BLT.18.218057

**Published:** 2018-11-06

**Authors:** Krycia Cowling, Elizabeth A Stuart, Roni A Neff, Daniel Magraw, Jon Vernick, Keshia Pollack Porter

**Affiliations:** a Department of Health Policy and Management, Bloomberg School of Public Health, Johns Hopkins University, 624 N. Broadway, Hampton House 380A, Baltimore, MD 21205, United States of America (USA).; b Department of Health Policy and Management, Bloomberg School of Public Health, Johns Hopkins University, Baltimore, USA.; c Center for a Livable Future, Department of Environmental Health & Engineering, Bloomberg School of Public Health, Johns Hopkins University, Baltimore, USA.; d Paul H. Nitze School of Advanced International Studies, Johns Hopkins University, Baltimore, USA.

## Abstract

**Objective:**

To investigate the relationship between joining the World Trade Organization (WTO) and the availability of several commodities with both harmful and protective effects for the development of noncommunicable diseases.

**Methods:**

We used a natural experiment design to compare trends in the domestic supply of tobacco, alcohol and seven food groups, between 1980 and 2013, in 21 countries or territories joining WTO after 1995 and 26 non-member countries, using propensity score weights. We applied a comparative interrupted time-series framework, by using multivariate random-effects linear models, adjusted for gross domestic product per capita, the percentages of urban population and female labour force participation. In the tobacco model, we controlled for Member States that had ratified the Framework Convention on Tobacco Control and in the alcohol model, the percentage of the population identifying themselves as Muslim.

**Findings:**

Following accession to WTO, member states experienced immediate increases in the domestic supply of fruits and vegetables of 55 g per person per day on average, compared to non-member countries. The analysis showed gradual increases in the geometric mean of the supply of tobacco and alcohol of 6.2% and 3.6% per year, respectively. We did not detect any significant changes in the availability of red meats and animal fats; seafood; nuts, seeds and legumes; starches; or edible oils; and results for sugars were inconsistent across model variations.

**Conclusion:**

The results suggest that WTO membership may lead to increases in both harmful and protective factors for noncommunicable disease, but further exploration of country-specific variation is warranted.

## Résumé

**Objectif:**

Étudier le lien entre l'adhésion à l'Organisation mondiale du commerce (OMC) et la disponibilité de différentes denrées avec des effets à la fois nocifs et protecteurs pour le développement des maladies non transmissibles.

**Méthodes:**

Nous avons utilisé un plan d'expérience dans les conditions naturelles pour comparer, entre 1980 et 2013, l'évolution de l'offre nationale de tabac, d'alcool et de sept groupes d'aliments dans 21 pays ou territoires devenus membres de l'OMC après 1995 et 26 pays non membres, à l'aide de pondérations par le score de propension. Nous avons appliqué un cadre comparatif de séries chronologiques interrompues, à l'aide de modèles linéaires à effets aléatoires multivariés, rajustés pour tenir compte du produit intérieur brut par habitant, du pourcentage de la population urbaine et de la participation des femmes à la vie active. Dans le modèle relatif au tabac, nous avons tenu compte des États membres ayant ratifié la Convention-cadre pour la lutte antitabac, tandis que dans le modèle relatif à l'alcool, nous avons tenu compte du pourcentage de la population s'identifiant comme étant de foi musulmane.

**Résultats:**

À la suite de leur adhésion à l'OMC, les États membres ont connu une augmentation immédiate de l'offre nationale de fruits et légumes de 55 g par personne et par jour en moyenne, par rapport aux pays non membres. L'analyse a révélé une augmentation progressive de la moyenne géométrique de l'offre de tabac et d'alcool de 6,2% et 3,6% par an, respectivement. Nous n'avons pas constaté de changements importants concernant la disponibilité de viandes rouges et graisses d'origine animale ; de produits de la mer ; de noix, graines et légumineuses ; de féculents ; ou d'huiles alimentaires ; et les résultats relatifs aux sucres variaient d'un modèle à l'autre.

**Conclusion:**

Les résultats suggèrent que l'adhésion à l'OMC peut entraîner une augmentation des facteurs à la fois nocifs et protecteurs pour les maladies non transmissibles, mais il convient de réaliser de nouvelles recherches sur les variations par pays.

## Resumen

**Objetivo:**

Investigar la relación entre la adhesión a la Organización Mundial del Comercio (OMC) y la disponibilidad de varios productos básicos con efectos tanto perjudiciales como protectores para el desarrollo de enfermedades no contagiosas.

**Métodos:**

Se utilizó un diseño de experimento natural para comparar las tendencias de la oferta interna de tabaco, alcohol y siete grupos de alimentos, entre 1980 y 2013, en 21 países o territorios que se incorporaron a la OMC después de 1995 y 26 países no miembros, mediante el uso de ponderaciones de propensión. Se aplicó un marco comparativo de series temporales interrumpidas, siguiendo modelos lineales de efectos aleatorios multivariados, ajustados según el producto interior bruto per cápita, los porcentajes de población urbana y la participación de la mujer en el mundo laboral. En el modelo del tabaco, controlamos a los Estados miembros que habían ratificado el Convenio Marco para el Control del Tabaco y, en el modelo del alcohol, el porcentaje de la población que se identificaba como musulmana.

**Resultados:**

Tras la adhesión a la OMC, los Estados miembros experimentaron un aumento inmediato de la oferta interna de frutas y hortalizas de 55 kg por persona y día de media, en comparación con los países no miembros. El análisis mostró incrementos graduales en la media geométrica de la oferta de tabaco y alcohol del 6,2 % y el 3,6 % anual, respectivamente. No se detectaron cambios significativos en la disponibilidad de carnes rojas y grasas animales, mariscos, nueces, semillas y legumbres, almidones o aceites comestibles, y los resultados para los azúcares fueron inconsistentes entre las variaciones del modelo.

**Conclusión:**

Los resultados sugieren que la pertenencia a la OMC puede dar lugar a un aumento de los factores perjudiciales y protectores de las enfermedades no contagiosas, pero es necesario seguir estudiando las variaciones específicas de cada país.

## ملخص

**الغرض:**

دراسة العلاقة بين الانضمام إلى منظمة التجارة العالمية (WTO) وتوافر العديد من السلع ذات التأثيرات الضارة والوقائية من أجل تطوير الأمراض غير المعدية.

**الطريقة:**

استخدمنا تصميم التجربة الطبيعية لمقارنة الاتجاهات في الإمداد المحلي للتبغ والكحول وسبع مجموعات غذائية، بين عامي 1980 و2013، في 21 دولة أو منطقة انضمت إلى منظمة التجارة العالمية بعد عام 1995 و26 دولة غير أعضاء، باستخدام أوزان درجات النزوع. طبقنا إطارًا زمنيًا متسلسلاً مقارنًا متقطعًا، باستخدام نماذج خطية ذات تأثيرات عشوائية متعددة، معدلة للناتج المحلي الإجمالي للفرد، والنسب المئوية لسكان الحضر ومشاركة المرأة في القوى العاملة. في نموذج التبغ، قمنا بالمكافحة في الدول الأعضاء التي صدقت على الاتفاقية الإطارية لمكافحة التبغ وفي نموذج الكحول، النسبة المئوية للسكان الذين يعرّفون أنفسهم بأنهم مسلمين.

**النتائج:**

بعد الانضمام إلى منظمة التجارة العالمية، شهدت الدول الأعضاء زيادات فورية في الإمداد المحلي من الفواكه والخضروات وصلت إلى 55 جم للشخص الواحد في اليوم ​​في المتوسط​​، مقارنة بالدول غير الأعضاء. أظهر التحليل زيادات تدريجية في المتوسط ​​الهندسي لإمدادات التبغ والكحول بنسبة 6.2٪ و 3.6٪ في السنة على الترتيب. لم نكتشف أي تغييرات جوهرية في توافر اللحوم الحمراء، أو الدهون الحيوانية، أو المأكولات البحرية، أو المكسرات، أو البذور والبقوليات، أو النشويات، أو الزيوت الصالحة للأكل، وكانت النتائج للسكريات غير متناسقة عبر تنوعات النموذج.

**الاستنتاج:**

تشير النتائج إلى أن عضوية منظمة التجارة العالمية قد تؤدي إلى زيادة في كل من العوامل الضارة والوقائية للأمراض غير المعدية، ولكن هناك تعزيز لمزيد من استكشاف الاختلافات الخاصة بكل بلد. ****

## 摘要

**目的:**

旨在调查加入世界贸易组织 (WTO) 与获取若干商品（此类商品对非传染性疾病的形成兼具有害因素和保护因素）之间的关系。

**方法:**

我们使用自然实验设计、倾向评分权重来比较 1980 年至 2013 年期间，在 1995 年之后加入 WTO 的 21 个国家或地区以及 26 个非成员国的烟草、酒精和七类食品组在各国内的供应趋势。我们采用断续时间序列框架，使用多变量随机效应线性模型，根据人均国内生产总值、城市人口百分比和女性劳动力参与进行调整。在烟草模型中，我们控制了已批准《烟草控制框架公约》的成员国，在酒精模型中，我们控制了自认为穆斯林国家所占的比例。

**结果:**

成员国加入世贸组织后，与非成员国相比，平均每人每天的水果和蔬菜国内供应量立即增长 55 克。分析显示，烟草和酒精每年的供应量以几何平均数的形式逐渐递增，分别为每年 6.2％ 和 3.6％。我们尚未发现获取红肉和动物脂肪有任何重大变化；海鲜；坚果、种子和豆类；淀粉；或食用油；以及与糖相关的结果在不同模型中是不一致的。

**结论:**

结果表明，成为 WTO 成员国可能会导致非传染性疾病的有害因素和保护因素增加，但仍需进一步探索其针对具体国家的不同变化。

## Резюме

**Цель:**

Членство во Всемирной торговой организации и изменения в факторах риска неинфекционных заболеваний: сравнительный анализ прерванных временных рядов, 1980–2013 гг.

**Методы:**

Авторы использовали естественный эксперимент для сравнения тенденций внутренних поставок табака, алкоголя и товаров семи продовольственных групп в период с 1980 по 2013 год в 21 стране или территории, которые вступили в ВТО после 1995 года, и в 26 странах, не являющихся членами этой организации. Анализ проводился с использованием весовых коэффициентов предрасположенности. Авторы применили методику сравнительных прерванных временных рядов и многовариантные линейные модели случайных воздействий, скорректированные по валовому внутреннему продукту на душу населения, процентной доле городского населения и участию женщин в трудовой деятельности. В модели для табака контролировались государства-члены ВТО, ратифицировавшие Рамочную конвенцию по борьбе против табака, а в модели для алкоголя — доля населения, идентифицирующая себя как мусульмане.

**Результаты:**

После вступления в ВТО в государствах-членах наблюдался немедленный прирост внутренних поставок фруктов и овощей (в среднем до 55 г в день на человека) по сравнению со странами, не являющимися ее членами. Анализ показал постепенное увеличение среднего геометрического значения поставок табака и алкоголя на 6,2 и 3,6% в год соответственно. Авторы не обнаружили существенных изменений в доступности мяса домашнего скота и животных жиров, морепродуктов, орехов, семян и бобовых, а также крахмала или пищевых масел. Что касается сахара, в разных моделях были получены несовпадающие результаты.

**Вывод:**

Результаты позволяют предположить, что членство в ВТО может привести к увеличению как полезных, так и вредных факторов распространения неинфекционных заболеваний, но необходимо проведение дальнейших исследований вариаций в различных странах.

## Introduction

Noncommunicable diseases are increasing in prevalence worldwide, especially in low- and middle-income countries, and now account for most of the global morbidity and mortality.^1^ Unhealthy food and alcohol consumption and tobacco use contribute to a significant proportion of the noncommunicable disease burden. These three risk factors collectively explain approximately one-quarter of the total disease burden worldwide.^2^ Evidence suggests that globalization and, in particular, trade and investment liberalization may play a key role in increasing the supply of these risk factors.^3^^,^^4^ Studies have shown that as countries liberalize, the consumption of unhealthy commodities increases.^5^^–^^7^ For example, consumption of meats high in fat has increased in the Federated States of Micronesia due to decades of foreign dependence and food imports,^8^ consumption of high-sugar and high-fat items has increased in Fiji after becoming increasingly reliant on food imports^9^ and meat and snacks consumption increased in Central America after lowering trade barriers.^10^

Few studies have used longitudinal data from many countries or causal inference methods to examine relationships between trade and investment liberalization and changes in noncommunicable disease risk factors, limiting conclusions about generalizability and causality from existing studies. A systematic review found that liberalizing trade and investment was associated with increased imports and consumption of edible oils, meats, processed foods and sugar-sweetened beverages, while the results for tobacco were inconclusive.^11^ A study examining 42 countries showed that between 1970 and 1995, higher trade volume was significantly associated with increased cigarette consumption in low- and middle-income countries only.^12^ However, another study did not detect any relationship between foreign direct investment and tobacco consumption in 50 low- and middle-income countries between 1997 and 2010. The study, though, found a significant positive association between increased foreign direct investment and consumption of alcohol and processed foods high in salt, fat and sugar.^5^ Case-control studies have identified an increase in sugar-sweetened beverage sales in Viet Nam following its accession to the World Trade Organization (WTO),^13^ while no significant changes were detected of such sales in Peru following its ratification of a free trade agreement with the United States of America.^14^

WTO agreements and institutions are an important set of trade policies. As of 2017, 164 countries were members of WTO, 126 of whom were original members of the predecessor General Agreement on Tariffs and Trade.^15^ Accession to the WTO is a discrete liberalizing event that is broadly comparable across countries, despite variations in accession commitments between countries, facilitating the comparison of countries joining the WTO with non-member countries. To provide quantitative evidence on the role of trade and investment liberalization in the global noncommunicable disease burden, we studied changes in the domestic supply of tobacco, alcohol and several food groups at the national level after WTO accession and compared to these trends in non-member countries.

## Methods

### Study design

We used a natural experiment approach to compare domestic supply patterns of nine commodities in 47 countries or territories, from 1980 to 2013: 21 countries or territories joining WTO between 1996 and 2008 (exposed group) and 26 countries not in WTO as of 2011 (unexposed group; Table 1). We defined exposure as accession to WTO and the post-exposure period was therefore the beginning of each country’s individual WTO joining date. The years 1980 to 1995 comprise the pre-exposure period for all countries, as the first countries joined the WTO in 1995.

**Table 1:** Countries and territories included in analysis, by WTO membership and domestic supply quantity for each commodity in 1993 and 2011

Country or territory	Commodity, by year																	
	Tobacco (g/capita older than 14 years)		Alcohol (kg/capita older than 14 years)		Fruits and vegetables (kg/capita)		Nuts, seeds and legumes (kg/capita)		Seafood (kg/capita)		Red meats and animal fats (kg/capita)		Starches (kg/capita)		Sugars (kg/capita)		Edible oils (kg/capita)	
	1993	2011	1993	2011	1993	2011	1993	2011	1993	2011	1993	2011	1993	2011	1993	2011	1993	2011
**WTO members (by WTO membership date)^a^**																		
Ecuador (21 Jan 1996)	321.4	517.5	28.4	55.7	181.9	141.5	5.0	3.7	7.2	8.5	25.9	40.6	187.6	155.2	26.4	20.1	14.0	15.3
Bulgaria (1 Dec 1996)	4263.5	2332.2	93.7	106.5	167.3	118.2	7.6	6.0	1.6	5.7	65.3	45.8	190.8	183.6	31.8	28.3	13.1	13.2
Mongolia (29 Jan 1997)	44.2	1468.9	6.6	48.1	11.7	76.9	0.6	1.8	0.1	0.7	109.6	85.9	113.5	187.0	12.7	14.5	0.3	6.7
Panama (6 Sept 1997)	640.3	1199.7	75.4	115.7	72.9	93.9	5.4	8.1	13.5	13.7	38.7	45.4	167.3	172.2	33.8	32.6	9.1	10.7
Kyrgyzstan (20 Dec 1998)^b^	366.6	2885.8	14.1	20.3	59.5	173.7	1.8	3.4	0.1	2.3	44.9	33.9	231.4	261.1	15.7	26.1	5.2	5.2
Latvia (10 Feb 1999)^b^	307.3	994.2	32.9	117.9	134.6	162.2	0.3	3.7	30.3	25.9	85.6	79.4	307.6	232.9	44.5	37.7	2.8	12.3
Estonia (13 Nov 1999)^b^	1388.2	1539.2	56.0	168.8	96.3	189.7	1.0	7.1	27.5	14.2	61.6	61.4	229.0	243.6	21.3	42.6	3.8	6.2
Jordan (11 Apr 2000)	2030.8	1679.4	3.1	1.2	153.3	188.2	10.5	12.2	3.9	5.7	16.4	13.8	162.8	204.0	40.7	41.2	15.2	21.7
Georgia (14 June 2000)^b^	268.7	2531.4	47.1	46.5	131.5	102.4	4.2	2.1	3.8	11.0	20.9	21.1	203.1	264.8	12.3	30.9	1.0	6.9
Albania (8 Sept 2000)	5851.6	1771.9	14.7	54.2	196.8	439.9	5.7	10.1	0.9	6.4	26.8	56.9	242.5	207.3	29.8	48.7	8.8	7.6
Oman (9 Nov 2000)	1093.0	2679.3	5.9	6.2	260.4	350.0	4.9	5.5	22.8	25.4	26.4	34.2	111.5	148.5	24.3	33.1	10.5	9.9
Lithuania (31 May 2001)^b^	927.2	371.6	51.9	165.7	131.2	149.7	2.1	4.3	24.3	43.1	67.0	68.2	279.8	249.6	29.5	45.2	4.6	9.7
Republic of Moldova (26 July 2001)^b^	9801.6	2766.3	56.3	57.1	188.4	133.4	4.7	2.3	0.5	11.1	34.4	26.3	253.1	180.3	23.9	21.2	4.8	10.5
China (11 Dec 2001)	3931.3	2766.6	24.5	54.6	155.3	437.8	8.2	10.9	14.5	34.0	28.1	50.7	236.8	225.0	5.2	6.9	5.4	7.9
Armenia (5 Feb 2003)^b^	2240.5	3054.4	15.6	10.0	136.4	390.7	0.0	2.2	1.2	3.2	21.0	37.1	241.1	188.6	23.0	36.8	0.4	7.5
Nepal (23 Apr 2004)	796.2	517.1	1.2	2.9	99.7	165.9	6.5	12.6	0.8	2.2	12.1	14.1	202.8	272.2	26.6	39.7	5.3	10.0
Cambodia (13 Oct 2004)	985.9	4148.3	2.1	34.2	68.9	65.1	2.2	12.1	7.4	40.6	15.5	15.4	192.2	206.0	4.5	24.5	2.9	1.9
Saudi Arabia (11 Dec 2005)	2624.2	2484.2	0.6	0.0	218.5	175.3	4.1	8.3	4.9	10.7	15.9	19.5	164.5	159.1	27.6	31.2	15.9	15.5
Viet Nam (11 Jan 2007)	533.9	1212.1	6.7	21.2	92.9	149.5	4.8	15.6	11.9	33.9	17.2	48.8	184.9	185.6	12.9	21.8	1.8	3.4
Ukraine (16 May 2008)^b^	1336.5	1402.3	37.2	93.7	131.1	223.4	6.0	3.7	7.6	14.3	53.7	35.2	333.7	282.7	45.6	48.9	9.4	12.9
Cabo Verde (23 July 2008)	336.1	287.3	40.7	62.8	85.9	202.9	5.6	13.7	14.4	12.1	31.3	21.4	188.4	177.2	19.0	20.9	9.2	8.5
**WTO non-member as of 2011**																		
Afghanistan	207.0	799.0	0.0	0.2	63.0	56.5	4.3	5.9	0.1	0.1	21.5	14.6	183.3	191.4	3.4	9.2	1.6	3.2
Algeria	1900.8	1250.2	4.5	6.0	119.1	254.9	5.5	9.9	3.7	4.1	14.4	15.2	250.4	287.8	27.5	28.1	16.5	15.5
Azerbaijan^b^	8924.3	2095.6	11.0	68.9	134.5	245.6	3.2	4.2	3.0	2.2	16.5	26.8	231.2	305.5	11.2	16.1	1.1	2.7
Bahamas	2008.1	1564.6	65.7	35.6	228.9	363.7	5.5	2.7	24.6	29.5	73.0	56.8	99.8	101.0	40.7	43.7	3.7	6.5
Belarus^b^	1420.8	2830.4	58.0	97.2	125.0	207.1	1.0	4.1	1.2	14.4	74.5	77.6	338.3	305.4	35.5	39.9	4.7	18.8
Democratic People's Republic of Korea	3606.3	4199.2	15.9	11.3	206.7	179.3	20.4	16.0	18.3	9.4	10.7	13.2	187.4	212.4	4.8	4.1	4.8	5.5
Ethiopia	174.6	116.5	7.3	16.4	17.6	26.5	10.4	20.5	0.1	0.3	8.4	9.4	169.7	210.6	3.7	6.5	1.3	3.1
French Polynesia	1838.6	1181.3	96.8	87.5	170.1	174.1	6.8	7.0	40.1	48.1	65.7	68.2	171.6	164.3	38.5	33.5	9.3	14.4
Iran (Islamic Republic of)	792.8	789.4	0.0	0.0	264.8	384.6	12.1	21.2	5.3	9.1	17.2	14.0	259.8	232.3	28.0	29.3	9.8	12.1
Iraq	1083.6	1676.5	6.6	2.9	213.6	155.1	4.8	3.5	1.3	2.9	7.8	5.2	177.4	187.1	20.2	18.9	13.4	17.3
Kazakhstan^b^	1689.4	1386.8	24.6	49.1	60.9	262.1	0.8	4.6	3.7	5.3	66.2	61.2	280.9	219.1	19.6	27.7	6.6	19.8
Kiribati	1105.0	2304.8	0.0	0.0	253.2	241.8	2.2	3.0	73.6	71.1	14.6	16.5	201.4	204.8	35.2	43.9	7.9	4.8
Lao People's Democratic Republic^c^	14 492.2	10 957.5	16.1	22.7	58.2	267.0	4.1	7.5	6.7	21.1	11.8	19.1	210.4	228.5	17.4	32.6	0.9	1.9
Lebanon	6887.7	5326.6	22.3	22.9	537.2	291.7	26.0	21.3	3.5	11.1	28.4	25.8	186.4	182.2	46.0	49.2	14.3	18.6
Liberia	382.0	249.1	15.3	16.1	92.0	61.9	6.5	3.6	4.8	4.4	8.9	8.0	246.6	260.3	19.5	17.1	17.1	17.4
New Caledonia	3558.9	2495.2	113.2	96.2	126.5	175.8	1.4	6.3	20.1	28.4	39.4	52.6	182.7	160.4	23.2	25.9	16.3	14.3
Russian Federation^b,d^	630.4	1834.8	47.1	107.4	108.2	179.6	2.8	3.7	14.3	22.4	68.2	55.7	282.3	261.3	35.5	49.0	6.7	13.2
Samoa^d^	3390.0	3072.2	60.7	52.7	268.8	369.2	0.9	4.6	41.6	47.4	53.3	41.8	105.2	203.5	24.8	30.2	3.3	7.3
Sao Tome and Principe	78.2	163.4	46.9	61.3	292.0	371.0	4.2	8.1	25.4	28.1	2.8	10.9	196.8	153.2	16.0	23.9	7.2	9.8
Sudan^e^	150.6	121.8	58.6	41.8	83.7	143.6	9.9	16.9	1.8	2.1	25.5	34.8	187.4	162.2	27.1	37.2	7.9	6.0
Tajikistan^b,c^	1605.8	148.7	8.8	1.4	146.6	185.7	1.4	4.0	0.5	0.5	13.0	12.4	189.1	175.6	10.9	19.0	11.4	10.7
Timor-Leste	283.0	922.3	6.2	7.9	46.6	49.8	10.8	12.6	0.0	6.0	40.2	32.8	305.5	218.3	3.3	11.5	1.1	4.7
Turkmenistan^b^	1361.6	1029.2	4.7	12.0	127.9	208.6	0.4	0.3	4.6	3.6	35.1	56.9	215.7	234.8	16.7	9.4	11.3	7.5
Uzbekistan^b^	53.4	287.8	11.4	18.6	160.0	311.6	0.7	1.2	0.9	0.7	27.0	37.9	232.4	235.1	13.4	10.2	13.3	10.2
Vanuatu^d^	578.9	336.2	11.1	6.7	341.7	277.3	6.2	7.4	31.3	33.7	35.5	31.4	243.7	311.1	9.2	20.3	7.0	5.7
Yemen	2104.4	2454.6	3.4	0.6	66.1	70.4	7.0	6.1	6.0	2.5	8.2	10.6	177.9	171.1	20.2	28.6	7.7	7.0

WTO: World Trade Organization.

^a^ We obtained membership dates from the WTO web site.^15^

^b^ We analysed former Soviet Union member states data from 1992.

^c^ We did not analyse data after 2012 since the country joined WTO in 2013.

^d^ We did not analyse data after 2011 since the country joined WTO in 2012.

^e^ Data ended in 2011 when country divided into Sudan and South Sudan.

Note: Quantities for each commodity for the periods before and after joining the WTO are presented for the first and last years with complete data for all countries, except for pre-exposure tobacco data for Oman, which are from 1992.

The commodities were tobacco (all types); alcohol (all types, including beer, wine and spirits); and seven food groups relevant to the development of noncommunicable diseases, either protective or harmful. These food groups were: fruits and vegetables; nuts, seeds and legumes; seafood; red meats and animal fats; sugars; starches; and edible oils. We based the selection of these food categories on a review of common elements of indices of dietary quality^16^^–^^19^ and dietary diversity^20^^,^^21^ and available evidence on the protective and harmful effects of major food groups for the development of noncommunicable diseases.^22^^–^^24^ A list of food items included in the different commodity groups and the data completeness for each item is available from the figshare data repository.^25^ We hypothesized that following WTO accession, the supply of tobacco, alcohol, edible oils, red meats and animal fats and sugars would increase; the supply of starches and nuts, seeds and legumes would decline. The expected trends in fruits and vegetables and seafood were unknown.

From our sample, we excluded original member states of WTO and all members of the former General Agreement on Tariffs and Trade. Nine countries in the unexposed group joined WTO in the final two years (2012–2013) or after the analysis period; data for these countries were censored to exclude values in or after the year they joined. For countries that comprised the former Soviet Union (eight exposed, seven unexposed), the analysis period begins in 1992, when independent countries were established.

### Data sources

The data sources for all commodities were the Food and Agriculture Organization national commodity balance sheets (tobacco) and food balance sheets (all other commodities), which measure the annual supply of each commodity, by country, and are widely used as a proxy for consumption.^26^^,^^27^ We obtained covariate data on urban population and female labour force participation from the World Bank’s World Development Indicators;^28^ population data from the United Nations Population Division;^29^ gross domestic product (GDP) per capita from the Institute for Health Metrics and Evaluation;^30^ percentage Muslim population from the Pew Research Center;^31^ and the ratification dates for the Framework Convention on Tobacco Control (FCTC) from the United Nations Treaty Collection.^32^

### Variables

We measured all commodity variables in units of grams (tobacco) or kilograms (all other commodities) per capita. For tobacco and alcohol, we restricted these measures to the population older than 14 years, as is standard.^33^^,^^34^ We controlled for the following key confounders established by the existing literature in all models: GDP per capita, urban population and female labour force participation.^4^^,^^5^ Models for alcohol included each country’s proportion of population identifying themselves as Muslim as a covariate, because being Muslim is linked to lower rates of alcohol use.^35^ Models for tobacco included a variable indicating whether the country had ratified the FCTC, because this ratification represents a commitment to reduce tobacco use.^36^

### Propensity score weights

With observational data, the non-random assignment of the exposure (in this case, WTO membership) can create imbalance in covariates and baseline levels of the outcome variables between the groups compared.^37^ Characteristics of the groups in the pre-exposure period are presented in Table 2. Although no differences were statistically significant, to improve comparability, we estimated and applied propensity score weights that optimized comparability on pre-exposure values of each commodity.

**Table 2:** Baseline characteristics of countries included in study on WTO membership and changes in noncommunicable disease risk factors

Covariates	WTO members^a^ (*n* = 21)	WTO non-members (*n* = 26)	Standardized difference in means (*P*)^b,c^
**No. of countries per area**			NA (0.55)
East Asia and Pacific	4	8	
Europe and central Asia	10	7	
Latin America and Caribbean	2	1	
Middle East and north Africa	3	5	
North America	0	0	
South Asia	1	1	
Sub-Saharan Africa	1	4	
**No. of former Soviet Union member states**	8	7	NA (0.41)
**Mean GDP per capita in 2005 Int$ (SD)**			
Year 1980	5565 (8 314)	6907 (9 697)	0.15 (0.69)
Year 1995	4805 (4 845)	6357 (11 005)	0.18 (0.55)
**Mean % of female labour force participation (SD)**			
Year 1980	44.1 (25.1)	42.1 (26.0)	−0.08 (0.82)
Year 1995	51.9 (18.3)	46.5 (22.4)	−0.26 (0.37)
**Mean % of urban population (SD)**			
Year 1980	38.2 (20.6)	37.2 (21.9)	−0.05 (0.90)
Year 1995	53.1 (20.1)	45.8 (20.9)	−0.35 (0.23)
**Mean % of Muslim population (SD)^d^**			
Year 1980	30.0 (40.4)	36.0 (43.4)	0.14 (0.70)
Year 1995	22.4 (36.2)	41.8 (42.8)	0.48 (0.11)
**Mean weight of commodity per capita^e^**			
Tobacco, gram (SD)^f^			
Year 1980	1890 (1 532)	2182 (1997)	0.16 (0.67)
Year 1995	1358 (1 045)	1913 (2 716)	0.26 (0.38)
Alcohol, kilogram (SD)^f^			
Year 1980	25.2 (36.9)	29.8 (33.0)	0.14 (0.72)
Year 1995	29.2 (26.2)	26.3 (27.5)	−0.11 (0.71)
Fruits and vegetables, kilogram (SD)			
Year 1980	108.9 (74.7)	165.8 (93.0)	0.64 (0.09)
Year 1995	137.1 (61.1)	159.0 (113.2)	0.23 (0.43)
Nuts, seeds and legumes, kilogram (SD)			
Year 1980	6.0 (3.5)	7.9 (6.2)	0.36 (0.34)
Year 1995	4.4 (2.6)	6.4 (6.7)	0.38 (0.19)
Seafood, kilogram (SD)			
Year 1980	7.7 (6.8)	17.0 (17.8)	0.62 (0.09)
Year 1995	9.5 (8.9)	13.9 (18.6)	0.30 (0.32)
Red meats and animal fats, kilogram (SD)			
Year 1980	28.0 (32.2)	27.2 (18.2)	−0.03 (0.93)
Year 1995	37.3 (23.8)	28.7 (20.9)	−0.38 (0.20)
Starches, kilogram (SD)			
Year 1980	193.1 (37.2)	223.0 (52.1)	0.62 (0.10)
Year 1995	215.5 (55.4)	207.8 (53.3)	−0.14 (0.63)
Sugars, kilogram (SD)			
Year 1980	24.4 (14.8)	23.9 (14.1)	−0.04 (0.93)
Year 1995	23.2 (8.9)	21.5 (13.1)	−0.15 (0.61)
Edible oils, kilogram (SD)			
Year 1980	6.1 (4.5)	6.8 (4.4)	0.15 (0.70)
Year 1995	7.3 (4.8)	8.3 (5.5)	0.19 (0.52)

GDP: gross domestic product; Int$: international dollars; NA: not applicable; SD: standard deviation; WTO: World Trade Organization.

^a^ Countries joining WTO between 1996 and 2008.

^b^ We calculated standardized difference in means as follows: (mean for non-member states – mean for member states)/(combined standard deviation).

^c^ For continuous variables, we used two-sided *t*-tests to calculate *P*-values. For or categorical variables, we used *χ^2^* tests.

^d^ Covariate used in alcohol models only.

^e^ Commodity available for domestic consumption.

^f^ Data for population older than 14 years.

Note: The years presented are the first (1980) and last (1995) years we used for analyses of the period before countries and territories included in the study started to join WTO.

In the first step, we estimated propensity scores to predict the probability of WTO membership as a function of annual values of each commodity in the pre-exposure period using a generalized boosted regression modelling approach.^38^^,^^39^ In the second step, we used propensity scores to construct weights for each country, with all exposed countries or territories receiving a weight of 1, and unexposed countries receiving a weight of *p*/(1−*p*), where *p* is the estimated propensity score. This weighting estimates the average treatment effect on the exposed group, i.e. the average effect of joining WTO for those countries or territories that did join.

**Fig. 1:**
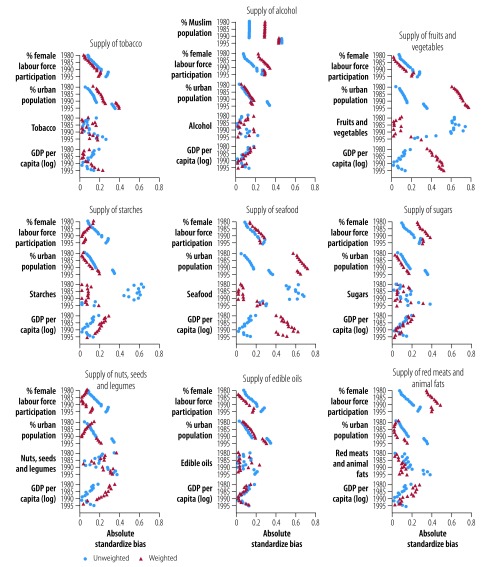
Unweighted and weighted absolute standardized biases for baseline commodity supply in countries included in the study, 1980–1995

Fig. 1 (available at: http://www.who.int/bulletin/volumes/97/1/18-218057) displays the balance, between the groups, for annual values of the commodities and covariates during the pre-exposure period, before and after applying weights. The balance metric is the absolute value of the difference in group means divided by the standard deviation across both groups; 0.25 is a generally accepted balance threshold.^37^ Improvements are reflected by the weighted values generally being closer to zero than unweighted values, though, in several cases, improving balance on commodities sacrificed balance on covariates. However, we further controlled for the influence of covariates in the regression models.

GDP: gross domestic product.

Notes: The period 1980–1995 represents the years before countries and territories start joining the World Trade Organization. Table 1 lists countries studied. We measured bias by the absolute standardized mean difference, that is: (mean for non-member states – mean for member states)/(combined standard deviation).

### Commodity models

We modelled changes in domestic supplies of the commodities using separate linear regression models for each of the nine commodities in a comparative interrupted time-series framework. We used WTO membership as the treatment (*t*) term and used a treatment*year interaction (*ty*) term to compare the pre- and post-exposure level and trend in the commodities (*c*), respectively, in the exposed versus unexposed groups.^40^ For unexposed countries, the WTO membership variable was always 0. For exposed countries, this variable ranged from 0 (before accession) to 1 (after accession); for the year of each country’s accession to WTO, we used a fraction reflecting the number of days of membership. Each commodity model had the following equation:
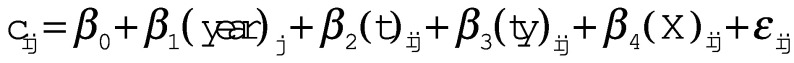
(1)where *i* indexes country; *j* indexes year (1980 to 2013); *x* is a set of countries- and year-specific covariates; *β*’s represent coefficients estimated by the linear model; and *ε* is the residual error term. Covariates for urban population, female labour force participation and percentage Muslim population (alcohol model only) were continuous, ranging from 0 to 100%. The FCTC covariate (tobacco model only) ranged from 0 (not ratified) to 1 (ratified), with a fraction reflecting the number of days after ratification in the year during which each country was ratified. All models were run with commodity-specific propensity score weights applied as inverse-probability-of-treatment weights.

We tested multiple model variations for each commodity. For six commodities (tobacco; alcohol; red meats and animal fats; seafood; nuts, seeds and legumes; and edible oils), we log-transformed the commodity values to constrain predicted values to be greater than 0. The key output of the best-performing model for each commodity is presented in Table 3; additional output and model fit graphs are available in the figshare repository.^25^

**Table 3:** Model output from best-performing model to study WTO membership and changes in noncommunicable disease risk factors, 1980–2013

Variable	Tobacco^a^	Alcohol^a^	Fruits and vegetables	Nuts, seeds and legumes^a^	Seafood^a^	Red meats and animal fats^a^	Starches	Sugars	Edible oils^a^
**Fixed effect, coefficient (*P*)**									
WTO membership	0.098 (0.477)	−0.118 (0.133)	19.794 (0.003)	0.107 (0.171)	−0.137 (0.436)	0.008 (0.865)	−6.277 (0.133)	−2.401 (0.115)	−0.070(0.296)
WTO membership*year	0.061 (0.054)	0.037 (0.050)	−1.276 (0.367)	−0.017 (0.151)	0.032 (0.367)	0.001 (0.875)	−0.120 (0.904)	0.250 (0.176)	0.005 (0.730)
GDP per capita^a,b^	0.449 (0.004)	0.496 (< 0.001)	7.571 (0.218)	0.313 (0.060)	0.826 (< 0.001)	0.184 (0.020)	5.308 (0.464)	6.133 (0.003)	0.150 (0.243)
% urban population	−0.017 (0.024)	0.014 (0.160)	1.993 (0.004)	−0.005 (0.491)	0.006 (0.637)	0.004 (0.533)	0.616 (0.189)	0.019 (0.879)	0.011 (0.052)
% female labour force participation	−0.010 (0.099)	−0.009 (0.202)	−1.029 (0.069)	−0.001 (0.804)	−0.036(0.016)	0.003 (0.553)	0.298 (0.371)	−0.133 (0.088)	−0.012 (0.102)
FCTC ratification^c^	−0.204 (0.032)	NA	NA	NA	NA	NA	NA	NA	NA
% Muslim population^d^	NA	−0.025 (< 0.001)	NA	NA	NA	NA	NA	NA	NA
Year^3e^	−9.72 × 10^−6^ (0.251)	NA	NA	NA	−1.46 × 10^−6^ (0.875)	NA	NA	NA	NA
Constant	4.759 (< 0.001)	−1.088 (0.337)	7.268 (0.883)	−1.413 (0.265)	−3.845 (0.022)	1.437 (0.049)	142.441 (0.020)	−18.940 (0.163)	0.066 (0.950)
**Random effects, variance (SE)**									
Intercept	0.944 (0.199)	1.660 (0.530)	6917.032 (1 656.286)	3.070 (1.585)	2.950 (0.747)	0.396 (0.073)	5228.010 (1 123.200)	87.917 (19.290)	8.46 × 10^−4^ (1.93 × 10^−4^)
Slope	7.83 × 10^−10^ (2.8 × 10^−10^)	0.003 (0.0012)	20.161 (5.345)	0.002(0.0013)	4.38 × 10^−10^ (9.97 × 10^−11^)	2.55 × 10^−4^ (5.61 × 10^−5^)	3.667 (0.828)	0.185 (0.0367)	0.953 (0.186)
Intercept and slope^f^	−1.3 × 10^−5^ (4.95 × 10^−6^)	−0.030 (0.013)	−255.959 (87.756)	−0.0713 (0.0449)	−1.13 × 10^−5^ (6.86 × 10^−6^)	−0.0058 (0.0015)	−93.382 (21.924)	−2.055 (0.632)	−0.0251 (0.0061)
Residual	0.215 (0.046)	0.071 (0.014)	437.215 (95.743)	0.076(0.0158)	0.162 (0.0454)	0.018 (0.0027)	184.153 (28.207)	12.292 (2.310)	0.052 (0.0107)

FCTC: Framework Convention on Tobacco Control; GDP: gross domestic product; NA: not applicable; SD: standard deviation; SE: standard error; WTO: World Trade Organization.

^a^ Natural logarithm of commodity values used in model.

^b^ In 2005 International dollars.

^c^ Only included in tobacco model.

^d^ Only included in alcohol model.

^e^ Coefficient values for individual year fixed effects not shown (when applicable); complete model output available from the figshare repository.^25^

^f^ Data presented are covariances and SEs.

### Sensitivity analyses

We did several sensitivity analyses to assess whether various aspects of the study design affected the estimated effects of WTO membership. First, to eliminate the influence of missing data, we restricted the analysis period to 1993 to 2011, years with complete data for all 47 countries. Second, because the effects of WTO accession may take time, we explored lagged values of the WTO membership and WTO membership*year terms. Third, to examine whether the effects of WTO membership were predominantly mediated through economic growth, we excluded GDP per capita from all models. Fourth, we excluded several countries in the unexposed group that may be poor comparisons due to war, famine or isolation from the global economy: Afghanistan, Democratic People's Republic of Korea, Ethiopia, Iraq and Sudan. Lastly, we stratified models by income group. All analyses were conducted in Stata version 14.2 (StataCorp. LCC, College Station, United States), except for the *twang* package for propensity scores, run in R version 3.3.2 (R Foundation, Vienna, Austria).

## Results

**Fig. 2:**
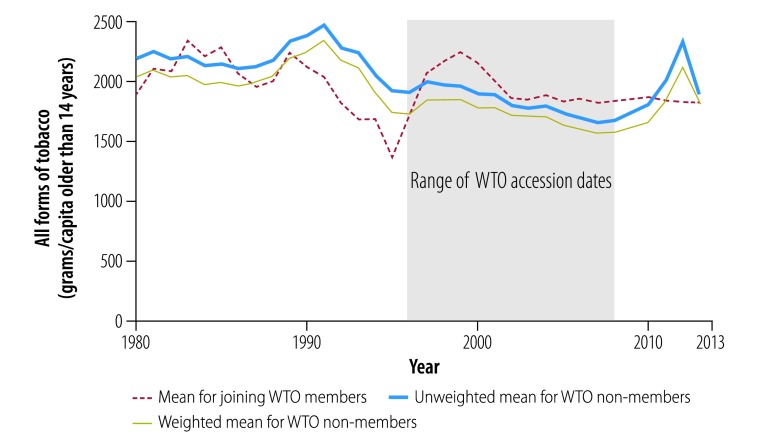
Changes in the supply of all forms of tobacco, by joining WTO members and non-member states, 1980–2013

**Fig. 3:**
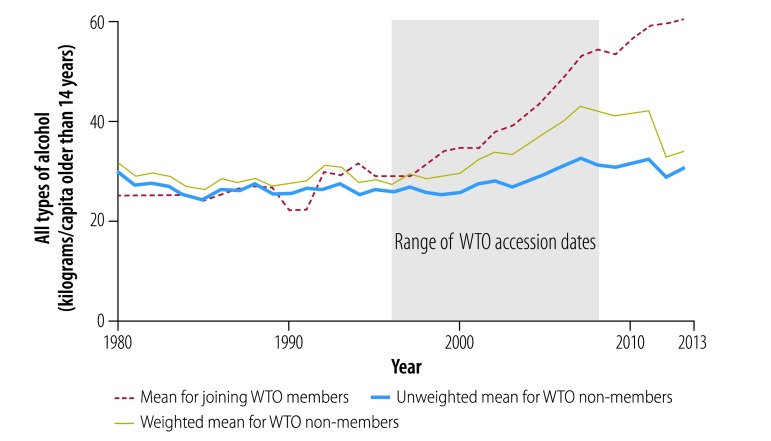
Changes in the supply of all types of alcohol, by joining WTO members and non-member states, 1980–2013

**Fig. 4:**
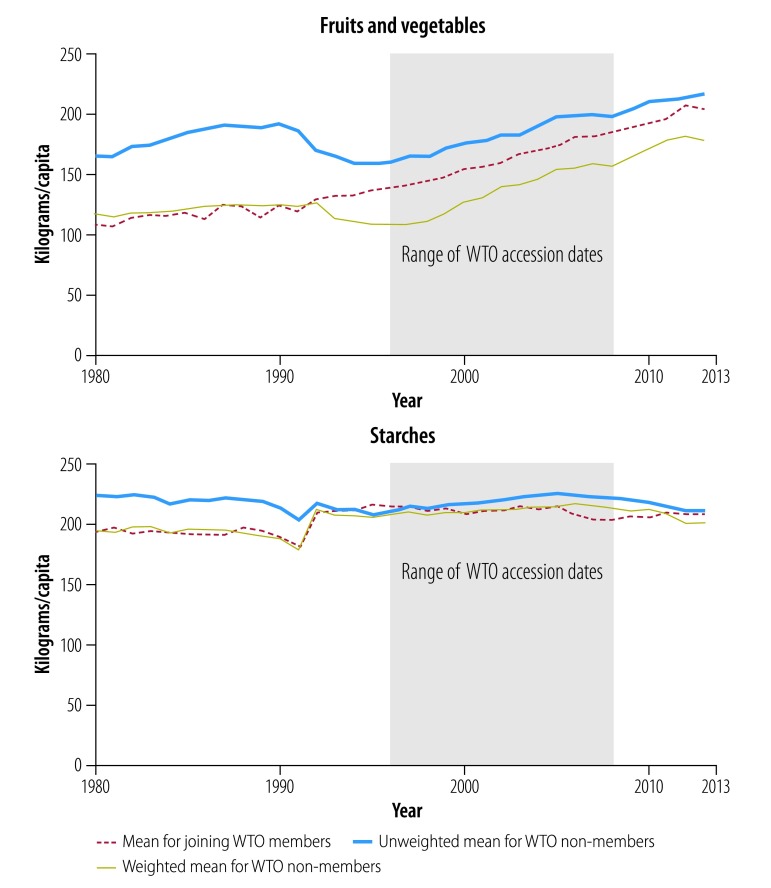
Changes in the supply of fruits and vegetables and starches, for joining WTO members and non-member states, 1980–2013

**Fig. 5:**
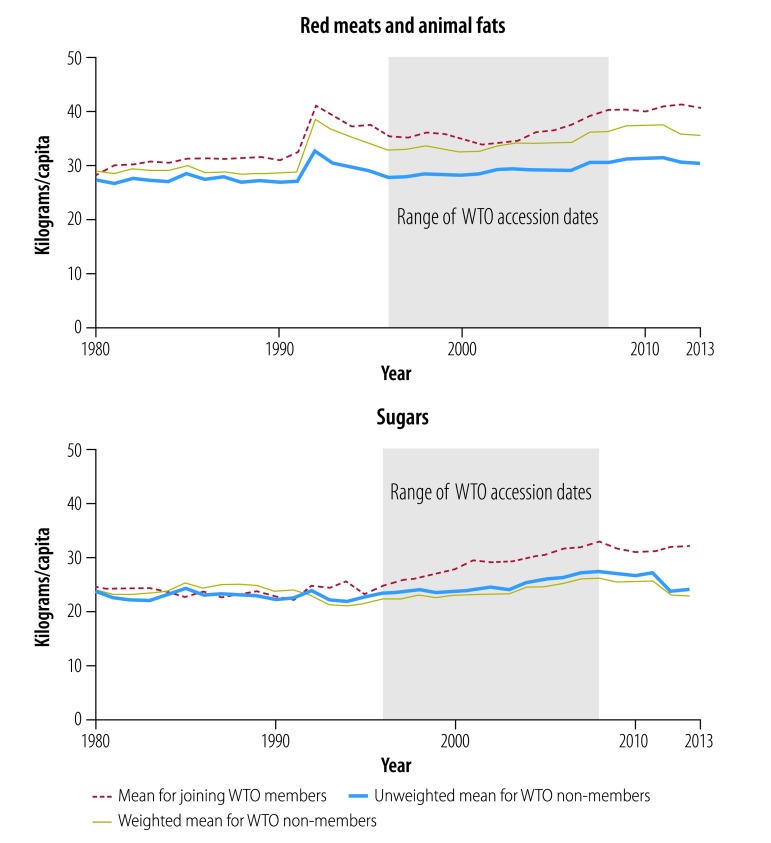
Changes in the supply of red meats and animal fats, and sugars, by joining WTO members and non-member states, 1980–2013

**Fig. 6:**
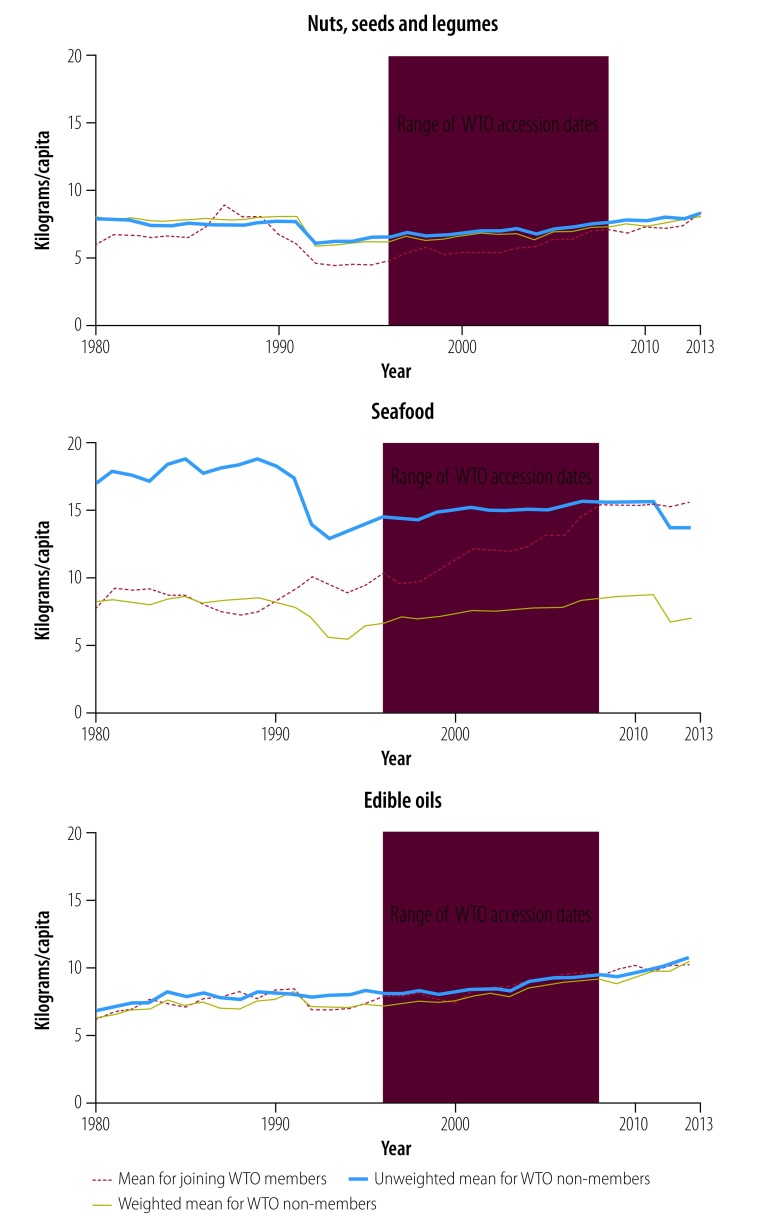
Changes in the supply of nuts, seeds and legumes; seafood; and edible oils, by joining WTO members and non-member states, 1980–2013

Fig. 2, Fig. 3, Fig. 4, Fig. 5 and Fig. 6 show average trends for each commodity for the exposed, unweighted unexposed and weighted unexposed groups. Trends during the pre-exposure period illustrate the improved comparability between the groups after weighting. Outputs from the best-performing models to estimate changes in supply of the commodities are shown in Table 3. The coefficients for the WTO membership and WTO membership*year terms indicate whether there is any difference in the level and trend, respectively, of each commodity for countries and territories joining the WTO, compared with non-WTO members. The domestic availability of fruits and vegetables increased the most: the average annual supply of fruits and vegetables was 19.79 kg per capita (95% confidence interval, CI: 6.60–32.99) higher in countries or territories that have joined WTO than in non-member countries. For tobacco and alcohol, the WTO membership*year coefficients suggest significant increasing trends in the availability of these products following WTO accession. The geometric means of the supply of tobacco increased by 6.2% (95% CI: 0.0–13.0) annually and of the supply of alcohol by 3.8% (95% CI: 0.0–7.7) annually. In the tobacco model, the FCTC ratification coefficient indicates an 18.5% (95% CI: 1.8–32.4) lower geometric mean supply of tobacco after ratification. In the random effect model, the intercept and slope are significantly different from zero for all commodities, indicating substantial remaining heterogeneity across countries in both the level and trend in domestic supply quantities (Table 3).

WTO: World Trade Organization.

Notes: The grey box represents the range of accession dates for countries and territories joining WTO. Aberrations in trends starting in 1992 likely reflect the changing composition of countries in each exposure group due to data availability for former Soviet Union countries. We obtained weighted means by applying tobacco-specific propensity score weights.

WTO: World Trade Organization.

Notes: The grey box represents the range of accession dates for countries and territories joining WTO. Aberrations in trends starting in 1992 likely reflect the changing composition of countries in each exposure group due to data availability for former Soviet Union countries. We obtained weighted means by applying alcohol-specific propensity score weights.

WTO: World Trade Organization.

Notes: The grey box represents the range of accession dates for countries and territories joining WTO. Aberrations in trends starting in 1992 likely reflect the changing composition of countries in each exposure group due to data availability for former Soviet Union countries. We obtained weighted means by applying commodity-specific propensity score weights.

WTO: World Trade Organization.

Notes: The grey box represents the range of accession dates for countries and territories joining WTO. Aberrations in trends starting in 1992 likely reflect the changing composition of countries in each exposure group due to data availability for former Soviet Union countries. We obtained weighted means by applying commodity-specific propensity score weights.

WTO: World Trade Organization.

Notes: The grey box represents the range of accession dates for countries and territories joining WTO. Aberrations in trends starting in 1992 likely reflect the changing composition of countries in each exposure group due to data availability for former Soviet Union countries. We obtained weighted means by applying commodity-specific propensity score weights.

The sensitivity analyses generally supported the main findings. The treatment effect on fruits and vegetables was robust in all sensitivity analyses. The trend coefficient for the alcohol supply stayed of a consistent magnitude and remained significant in most analyses. The trend coefficient for the tobacco supply also remained of a consistent magnitude, but not always statistically significant. In only the lagged effect models, treatment effects for sugars were significant and similar in magnitude to those in the main model, providing some evidence of an initial decrease in the availability of sugars following WTO accession, followed by a minimal steady increase. When we stratified the analyses by country income group, the results did not support any of the main conclusions. However, propensity score weights were generated to balance the sample and likely generated false results from these models, which were run with 23 countries or less (out of the total 47) per income group. Further details on each sensitivity analysis are available in the figshare repository.^25^

## Discussion

Here we show that following a country’s accession to WTO, there was a significant increasing trend in the domestic supply of alcohol; a borderline significant increasing trend in the supply of tobacco; and a significant immediate increase in the availability of fruits and vegetables, compared with non-member countries. Assuming that increases in supply likely translate to increases in consumption, these changes have both positive and negative implications for global health. For example, recent research has indicated that any amount of alcohol consumption increases the risk of a range of negative health outcomes, hence an increase in alcohol supply could be harmful.^41^ Likewise, an increase in tobacco use is negative, as tobacco contributes to several noncommunicable diseases.^42^ In contrast, increases in fruit and vegetable consumption can protect against the development of numerous noncommunicable diseases.^24^ The WHO recommends a 400 g intake of fruit and vegetables daily,^43^ although even an intake of 200 g per day has been found to reduce the risk of many noncommunicable diseases and premature mortality.^44^ We estimate that average increase in the supply is about 55 g of fruits and vegetables per person per day higher in the countries after joining WTO.

Our results provide more evidence about the links between trade liberalization and global health, suggesting that trade agreements should be considered as social determinants of health at the global scale. As the burden of noncommunicable diseases continues to grow, stakeholders should prioritize identifying the most effective strategies to curb the increase in risk factors, including tobacco and alcohol use and poor diet. For example, addressing aspects of trade and investment policies that alter the supply of these products can help to tackle the noncommunicable disease burden at the root cause level. This approach can be achieved through actions grounded in the Health in All Policies framework^45^ and the application of health impact assessment to proposed trade and investment policies.^46^ At the global level, further development and consideration of international agreements to prevent and control noncommunicable diseases, like the FCTC, but focused on other noncommunicable disease risk factors, is warranted. Such agreements can legally bind countries to health commitments, providing a counterweight for commitments to international trade and investment rules.

Our findings are suggestive but not conclusive, warranting additional exploration and we suggest several potential avenues for future research. For example, given substantial unexplained country-specific heterogeneity indicated by country random effects in all models, additional analyses of smaller groups of countries and individual countries are needed. The effects of WTO membership may differ by level of economic development and other country-specific factors, such as geography and climate, which affect the baseline supply of various food groups, tobacco and types of alcohol. Researchers could recreate this analysis with one income group at a time and a similar analysis of selected low- and middle-income countries with substantial noncommunicable disease burdens may provide further insight into the links between WTO membership and changes in noncommunicable disease risk factors. Single country studies could permit more nuanced understanding, for example, by looking at tariff changes for specific products and subsequent changes in their supply. Another area for future studies is to examine differences associated with specific provisions in WTO accession agreements and other trade and investment policies, as specific concessions differ. Understanding which components of these treaties have the greatest influence on noncommunicable disease risk factors and other aspects of public health is important when tailoring future agreements to be more health-promoting. Further, researchers should also conduct similar analyses for other products that contribute to noncommunicable diseases, particularly unhealthy foods high in fat, salt and sugar.

This study both supports and contradicts findings from previous research. We were not able to confirm findings showing increases in consumption of meat^10^ and edible oils^47^ following trade liberalization. Discrepancies may be due to differences in the sample; previous studies examined only one to five countries in the same geographical region. Studies showing trade-related increases in sugar-sweetened beverage consumption^13^^,^^48^ are somewhat supported by our weak finding that the domestic supply of sugars increases steadily over time following WTO accession. Our results confirm previous findings of increased tobacco^7^^,^^12^ and alcohol^5^ consumption associated with trade liberalization. Finally, few studies have examined fruits and vegetables consumption in the context of trade liberalization, but our findings support the results of an analysis showing that changes in trade policies led to an increase in imported fruit in five Central American countries.^10^

This study has limitations. A primary limitation is the comparability of countries joining versus not joining WTO. We assumed that trends would have been similar between the two groups if none of the countries joined WTO. However, differences in trends could be due to the influence of other unobserved events correlated with WTO accession and the outcomes of interest or innate characteristics of countries in either group.

Another key limitation is the quality of the commodity data, which measure the available supply of each commodity and are only a proxy for consumption, the true measure of importance for health. In addition, there was substantial missing data for certain items summed to create commodity variables, which may affect the validity of the data for these categories. The commodities analysed were also limited by the available product categories; importantly, this did not allow us to distinguish changes in trends in the availability of specific foods high in fat, salt or sugar, an important determinant of obesity and noncommunicable diseases.^49^ Finally, for tobacco and alcohol, illicit sales and homemade varieties are not captured in this data, which may comprise substantial portions of supply and consumption in certain countries.

In conclusion, changes in domestic supply of alcohol, tobacco and fruits and vegetables could have important implications for public health, particularly for the development and prevention of noncommunicable diseases. Overall, findings indicated substantial country-level heterogeneity. Therefore, additional exploration of variations across countries is critical to identify factors that mitigate the negative role and enhance the positive role of trade and investment agreements in the global noncommunicable disease burden.
